# Metalloproteinases as Biomarkers and Sociomarkers in Human Health and Disease

**DOI:** 10.3390/biom14010096

**Published:** 2024-01-11

**Authors:** Davide Costa, Enrica Scalise, Nicola Ielapi, Umberto Marcello Bracale, Michele Andreucci, Raffaele Serra

**Affiliations:** 1 Department of Medical and Surgical Sciences, Magna Graecia University of Catanzaro, 88100 Catanzaro, Italy; davide.costa@unicz.it (D.C.); enrica.scalise@unicz.it (E.S.); 2 Interuniversity Center of Phlebolymphology (CIFL), Magna Graecia University of Catanzaro, 88100 Catanzaro, Italy; 3 Department of Public Health and Infectious Disease, “Sapienza” University of Rome, 00185 Rome, Italy; nicola.ielapi@uniroma1.it; 4 Department of Public Health, University Federico II of Naples, 80131 Naples, Italy; umbertomarcello.bracale@unina.it; 5 Department of Health Sciences, Magna Graecia University of Catanzaro, 88100 Catanzaro, Italy

**Keywords:** matrix metalloproteinases, psychosocial aspects, complexity science, complexity paradigm, chronic diseases, health, well-being, interdisciplinary approach, biomarkers, sociomarkers

## Abstract

Metalloproteinases (MPs) are zinc-dependent enzymes with proteolytic activity and a variety of functions in the pathophysiology of human diseases. The main objectives of this review are to analyze a specific family of MPs, the matrix metalloproteinases (MMPs), in the most common chronic and complex diseases that affect patients’ social lives and to better understand the nature of the associations between MMPs and the psychosocial environment. In accordance with the PRISMA extension for a scoping review, an examination was carried out. A collection of 24 studies was analyzed, focusing on the molecular mechanisms of MMP and their connection to the manifestation of social aspects in human disease. The complexity of the relationship between MMP and social problems is presented via an interdisciplinary approach based on complexity paradigm as a new approach for conceptualizing knowledge in health research. Finally, two implications emerge from the study: first, the psychosocial states of individuals have a profound impact on their overall health and disease conditions, which implies the importance of adopting a holistic perspective on human well-being, encompassing both physical and psychosocial aspects. Second, the use of MPs as biomarkers may provide physicians with valuable tools for a better understanding of disease when used in conjunction with “sociomarkers” to develop mathematical predictive models.

## 1. Introduction

Proteases are important enzymes that are involved in many aspects of biology, and proteolysis plays a key role in protein post-translational modifications. The 473 human proteases are divided into five classes of catalysis as reported in the MEROPS database: metallo (148), serine (152), cysteine (136), aspartic acid (21), and threonine proteases. The main function of proteases is to cleave or degrade all proteins [1,2,3,4]. Metalloproteinases (MPs) are a group of multidomain zinc-dependent endopeptidases named metzincin proteases, which are divided into six families: astacins (an enzyme found in various bacterial species and humans), adamalysins or ADAMS (consisting of a disintegrin and metalloproteinase), ADAMTS (consisting of a disintegrin and metalloproteinase with thrombospondin motif), pappalysin (pregnancy-associated plasma protein), serralysin (bacterial enzyme), and matrix metalloproteinases (MMP) or matrixin [4,5,6,7]. The name “metzincins” originates from the fact that these enzymes possess a residue of methionine (Met) at their active site and require the presence of zinc to carry out their enzymatic reactions [8]. The primary structures of the metalloproteinase domains have a high heterogeneity among the families, while the overall folding of the proteins is similar [9]. In recent research, there has been a predominant focus on metalloproteinases, specifically the MMP and ADAM families of proteinases. This is mainly due to their importance in pericellular proteolysis and their involvement in human diseases, including cancer and immunological changes [10]. Matrix metalloproteinases (MMPs) have attracted considerable interest, with the first studies dating back to 1962 when J. Frederick Woessner’s pioneering research on these enzymes revealed their ability to degrade collagen in the mammalian uterus [11]. Jerome Gross and Charles Lappiere observed collagen turnover in anauran tadpoles, which was marked by collagen breakdown in the skin, gills, and gut during metamorphosis [12]. The initial MMP, known as “tadpole collagenase” (MMP-1), was isolated from the tail and back skin of a tadpole in 1966 [13]. In 1988, MMP-2 was sequenced and given the name 72-kDa type IV collagenase/gelatinase A, as it demonstrated the ability to break down denatured collagen/gelatin [14]. In 1985, MMP-3, initially referred to as proteoglycanase due to its ability to degrade proteoglycan and casein, was purified from rabbit synovial fibroblasts and later named stromelysin [15,16]. In 1991, researchers studied seven MMPs (MMP-1, -2, -3, -7, -8, -9, and -10), and once the Human Genome Project was finished, it was discovered that in humans, the MMPs were composed of 23 members with various domains [17,18]. MMPs, or matrix metalloproteinases, consist of several components: an 80-amino acid propeptide, a 170-amino acid catalytic metalloproteinase domain, a linker peptide of varying lengths known as the “hinge region,” and a 200-amino acid hemopexin (Hpx) domain. The organization of these domains and their substrate preferences determine the different types of MMPs, including collagenases, gelatinases, matrylisins, and membrane-type (MT) MMPs [19,20]. MMPs, as their names suggest, have the ability to break down and deteriorate proteins of the extracellular matrix. However, research indicates that only 31% of the proteins they target are ECM proteins, while the remaining 69% are non-ECM proteins [21]. It is important to note that the ECM is a structural component found in all tissues throughout the body [22]. Using the complete genome sequences, scientists have successfully identified more than 300 ECM proteins found in mammals that make up the fundamental matrisome. This includes a variety of components, such as collagens, proteoglycans, glycoproteins, and ECM-associated proteins. There are two primary types of ECM: the interstitial connective tissue matrix and the basement membrane (BM) [23]. The former provides structural support to tissues, while the latter acts as a barrier between the epithelium and neighboring stroma [24]. The ECM is in a constant state of remodeling, degradation, and processing to maintain tissue homeostasis. These dynamic processes have a profound impact on the behavior of cells and tissues [25,26]. The ECM regulates basic biologic functions: cell movement, shape, growth, survival via cell adhesion, and cell-ECM and cell–cell interactions [5]. The interaction between epithelial cells and the components of the extracellular matrix (ECM), which act as ligands for membrane receptors, leads to the activation of cellular processes such as proliferation, apoptosis, adhesion, migration, survival, and differentiation [24]. Cleavage of ECM components, facilitated by proteases, leads to the release of bioactive molecules such as growth factors [21]. MMPs, ADAMs, ADAMTSs, plasmin, cathepsins, and other proteases are involved in the regulation of ECM remodeling [26,27,28].

For example, active MMP-11 is released by fibroblasts after hormonal stimulation and promotes epithelial cell apoptosis and growth of connective tissues [29,30,31]. The remodeling of the extracellular matrix (ECM) is also influenced by the downregulation of protease inhibitors such as tissue inhibitors of metalloproteinases (TIMPs), cystatins, and serpins [26]. Within the extracellular matrix (ECM), the degradation and processing of growth factors and their receptors are facilitated by MMPs. These factors participate in the modulation of angiogenesis in the context of wound healing [32]. In fact, MMPs have the ability to activate VEGF, a key regulator of angiogenesis that promotes vascular permeability and blood vessel growth [33,34]. Numerous non-ECM substrates have been extensively researched, including chemokines, cytokines, cell surface receptors, growth factors, metabolic proteins, and nuclear proteins. The regulation of MMPs plays a crucial role in determining the specific substrates on which they cleave. Substrate processing is dependent on the type of cell, tissue, and disease [35,36,37]. Various studies have highlighted the significant role of MMPs in modulating the biological functions of chemokines. This modulation can occur via activation or inactivation cleavage, resulting in a positive or negative signal transmitted via the downstream signaling of the chemokine receptor [37,38,39,40]. The presence of intracellular MMP2 within cardiac myocytes was discovered by Wang et al. [41] in 2002, indicating that MMPs play a role beyond the degradation of ECM substrates. In the case of human liver cancer cells (HepG2 cells), MMP3 was observed to be located in the nucleus, and its catalytic activity was shown to induce apoptosis via experimental studies. Although this study demonstrates the intracellular role of MMPs, the mechanism of their action in cells remains unknown [42].

Membrane shedding is a process in which bioactive protein domains are released, and cellular responsiveness to growth factors and cytokines is altered. MMPs, including membrane-type MMPs and soluble MMPs, play a role in this shedding mechanism [5]. Additionally, MMPs have the ability to modify the activity of other proteases. For example, MT1-MMP activates MMP-2, which in turn cleaves cystatin C and secretory leukocyte protease inhibitor [43,44,45].

Understanding the biology of proteases and the role of MMPs in pathophysiology is based largely on knowledge of their substrates [46]. In a study conducted by Rodriguez et al. [47], a comprehensive categorization of MMP substrates was presented based on their respective pathophysiological functions. These functions encompassed various areas such as extracellular matrix and bone remodeling, blood vessel development and angiogenesis, cell migration and proliferation, invasion and metastasis, tumor growth and apoptosis, inflammation, innate immunity, and wound healing. The relationship between MMP expression and the development of diseases indicates the physiological importance of MMPs. Increased levels of MMP have been observed in various human pathologies, such as cardiovascular disease, rheumatoid arthritis, neurological disorders, and cancer [48,49,50,51,52,53]. The function of MMPs in disease progression and resolution is not straightforward, as their functions can transition from being destructive enzymes to serving as cell signaling regulators and disease antitargets. This suggests that MMPs have a complex and multifaceted involvement in the etiology and progression of diseases, surpassing initial assumptions [47,54,55]. MMPs have been studied in the context of cancer progression for their ability to remodel BM and increase metastasis [37,56,57]; however, Dufour and Overall demonstrated that at least 10 MMPs play a protective role in cancer [35]. Neurological disorders that are typical of the central nervous system (CNS), including neuroinflammation, multiple sclerosis, and malignant gliomas, exhibit a common trait: the release of MMPs by both CNS resident cells and immune cells infiltrating the system. These MMPs contribute to the breakdown of the blood–brain barrier, facilitate leukocyte recruitment, and regulate the signaling of cytokines and chemokines [58,59,60]. Although numerous studies have proposed that inhibiting MMPs may provide neuroprotection, certain MMPs may actually play a role in limiting neuroinflammation or promoting tissue repair [36]. When considering inflammatory diseases, it is crucial to acknowledge that MMPs play a dual role—they are not only linked to extracellular matrix breakdown and tissue damage but also have a protective function, specifically MMP-2, -8, and -12 [61,62,63]. Studies on atherosclerotic plaque stability demonstrate that, using an APOE/MMP double knockout mice, MMP-3 and MMP-9 play a protective role in reducing plaque growth and improving plaque stability, while MMP-12, on the other hand, promotes lesion expansion and destabilization [64]. These studies demonstrate the diverse functions of MMPs in the development of human diseases. Despite more than six decades of research, there are still intriguing mysteries surrounding the biological roles of MMPs that need to be unraveled [17,64]. We aim to contribute to understanding these unresolved aspects, recognizing the significant impact of pathologies on patients’ lives and their influence on disease progression [65]. It is very difficult to remain confined to a biological dimension without analyzing the psychosocial implications in affected individuals; therefore, these problems must also be evaluated to obtain a complete vision of MMPs between health and disease [65]. It is now widely accepted that organisms are constantly immersed in an environment that provides them with information. Similarly, understanding that complex phenomena can be understood by studying the ability of living systems to self-organize in response to external stimuli has become commonplace [66]. These concepts cannot be accounted for by linear, mechanistic interactions among the individual components of a system. Instead, self-organization and complexity are fundamental to understanding the functioning of integrated systems, whether in the fields of physics, chemistry, biology, or engineering [67].

During the 1960s and 1970s, Edgar Morin was among the pioneering thinkers who introduced a new perspective on complexity, challenging the deterministic and reductionist view of science. This paradigm shift highlights the significance of nonlinearity, disequilibrium, disorder, and interaction [66]. Morin argues that the world is predominantly composed of open systems characterized by constant flux and disorder, which pose threats to all living systems while also providing nourishment. In contrast to classical science, which prioritizes a strictly objective approach by eliminating subjectivity, complexity theory emphasizes the role of the observer and the subject in relation to the object [66,68]. In Morin’s perspective, complexity acknowledges the interconnectedness of human beings with the natural world, rejecting the notion of isolating humans conceptually from nature. This viewpoint enables the establishment of new connections between biology, anthropology, and sociology, as it recognizes the existence of underlying links between seemingly unrelated aspects of human life. Living systems, characterized by complexity, disorder, and self-organization, possess the ability to respond to environmental stimuli in various combinations that are not predetermined by genetic material. The interaction of open systems facilitates the interplay between biological and cultural evolution, intertwining these processes [66,69,70]. Taking into account the external environment as a crucial factor in the interpretation of information by living beings, the effects of MMPs on human diseases can be explained. This perspective acknowledges the inseparability of external influences from life itself [65,71]. The intricate interplay between genetics, environment, culture, and individual and species characteristics is facilitated by the human brain, which acts as a central integrator connecting the biological, cultural, and spiritual realms [66]. These concepts prompt us to examine the holistic connection between MMP, disease, and social dimensions of existence, urging the pursuit of interdisciplinary research.

The aim of this scoping review is to clarify the role that MMPs play in the development and progression of neuropsychiatric diseases, cardiovascular diseases, rheumatoid arthritis, chronic wounds, periodontal disease, complex pathologies that have a strong impact on the social and psychological sphere of those affected. Our aim is to search the scientific literature for articles that place MMPs involved in the above-mentioned diseases in a psychosocial context, according to the “complexity paradigm” theorized by Edgar Morin.

## 2. Materials and Methods

### 2.1. Study Design

The review adhered to the guidelines of the Preferred Reporting Items for Systematic Reviews and Meta-Analyses, in particular the extension for scoping reviews (PRISMA-ScR) [72], and followed the methodological framework for conducting scoping studies established by Arksey and O’Malley [73].

### 2.2. Search Strategy

A systematic literature review was conducted using the following Medline (via PubMed), Scopus, and Web of Science electronic databases, with search term combinations adapted to the syntax and functionality of each database. There is no date limit for the search. The search query used is shown in Table 1.

### 2.3. Inclusion and Exclusion Criteria

The gray literature was then identified via a targeted search up to page 20 using the Google Scholar search engine [74]. Only documents written in English were eligible for inclusion.

The criteria for inclusion/exclusion of the article were established using a modified PICOTS (population, intervention, comparator, outcome, time, and setting) framework [75] and are described in Table 2.

### 2.4. Study Selection

Three independent authors reviewed the title list to create a preliminary classification list of retrieved articles. These articles were subjected to abstract review, the full text of potentially relevant articles was obtained, and their eligibility for inclusion was assessed. The authors then searched the references of previously selected articles to identify additional relevant studies. Any disagreements regarding the inclusion of articles were resolved by discussion among the three authors.

### 2.5. Data Extraction and Synthesis

Data extracted from the articles included various elements such as authors, titles, years, countries, methods/study design, population, and study content. To ensure the extraction of all potentially relevant data, the data sheets were meticulously organized. The filtering process was carried out independently by both the author and a member of the review team, and the results were then reviewed by another author to guarantee accuracy in the selection process. The extracted form contained the following elements: reference, title, journal, publication year, type of study, aim/research questions, country, and key findings.

Where data were missing, efforts were made to obtain additional information by contacting the authors of this study. The results are presented in a narrative format since a comprehensive analysis determined that this was the most effective approach to present the many findings of the included studies. An evaluation of the bias or methodological quality of the articles was not considered, as this was not a requirement to conduct a scoping review [72].

## 3. Results

### 3.1. Selected Studies

**Figure 1:**
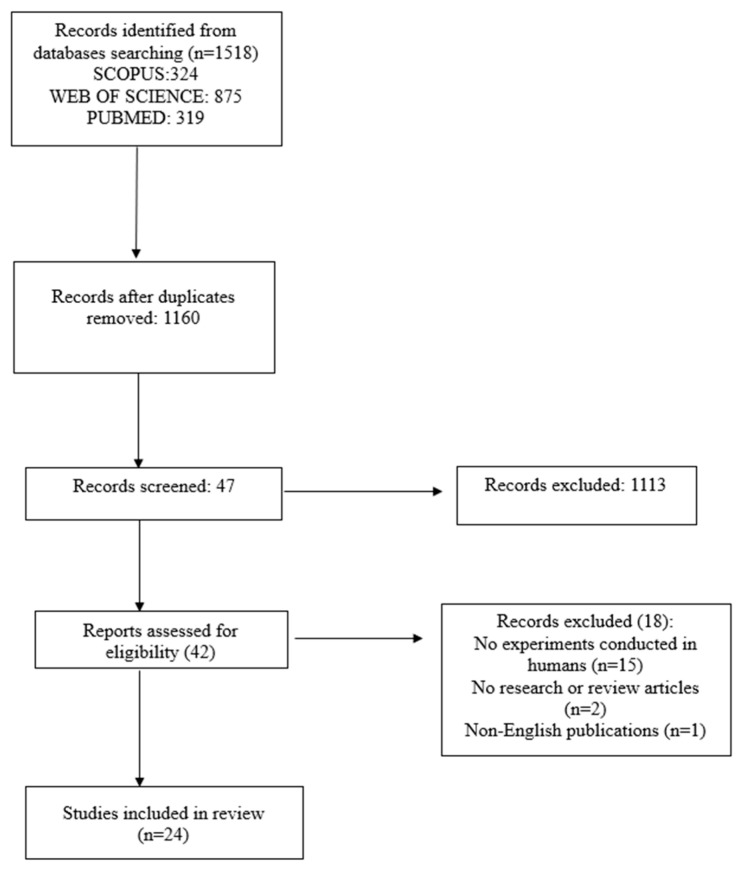
PRISMA-ScR 2018 flow diagram.

We initially identified a total of 1518 articles from various databases, including 324 from Scopus, 875 from Web of Science, and 319 from PubMed. After removing duplicates (n = 358), we were left with 1160 articles to screen. Throughout the screening phase, 1113 articles were excluded, leaving us with 47 articles that were evaluated for eligibility. Of these, 18 articles did not meet the inclusion criteria and were excluded, resulting in the final inclusion of 24 articles for review. For a visual representation of the article selection process, please refer to Figure 1.

### 3.2. General Characteristics of the Included Studies

This review covers a total of 24 articles, with Table 3 providing a comprehensive summary of the general characteristics found within the included studies.

### 3.3. Temporal Extension of the Included Studies

**Figure 2:**
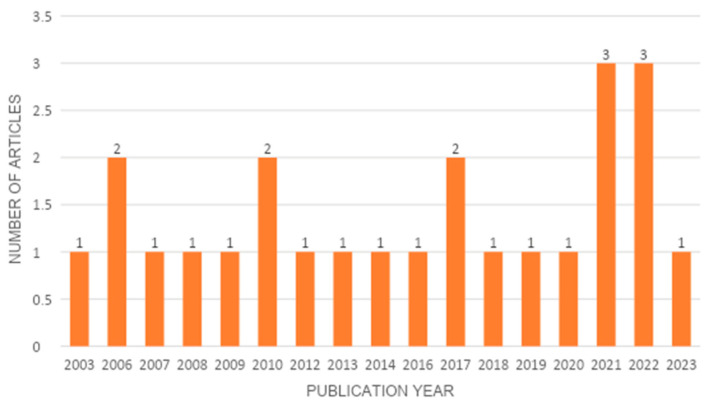
The publication years of the papers.

Within the compilation of the current analysis, the researchers discovered papers that span various years. A visual representation of the years of publication of these articles can be seen in Figure 2, which provides a comprehensive breakdown of the details.

### 3.4. Distribution of the Journals of the Included Studies and Their Research Areas

Based on the examination of 24 separate papers published in various journals, it is evident that both MMPs and social aspects are integral components of research in diverse fields, ranging from biology to psychiatry. Most of these journals fall within the realms of psychiatry, oncology, or biology. In Table 4, the journal distribution and research areas of the included studies are quoted.

### 3.5. Geospatial Distribution of Included Studies

**Figure 3:**
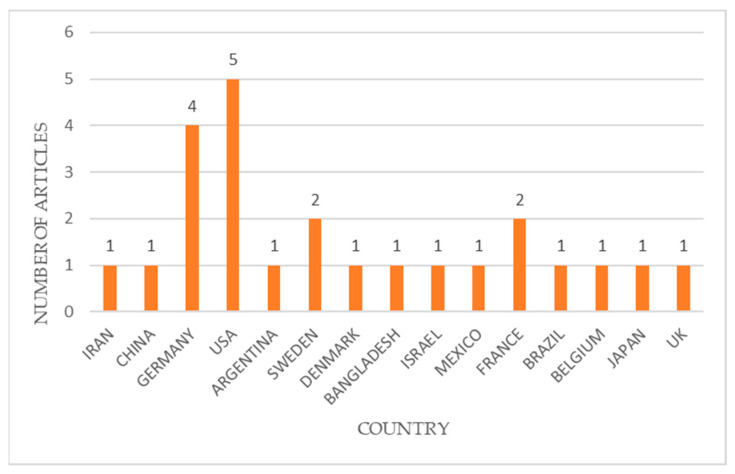
Geospatial distribution of papers included.

A geospatial analysis of the included papers showed that 24 studies were conducted in the context of 15 countries, of which most of the studies were conducted mainly in the United States (5 articles); Germany (4 articles); Sweden and France (2 articles); followed by India, Africa, Brazil, European countries, China, etc. (only 1 article). Figure 3 shows the details of the geospatial distribution of the included papers.

To facilitate the understanding of the scientific articles presented in the previous section, the issues related to the relationship between matrix metalloproteinases and psychosocial aspects of patients were grouped according to the main pathologies of interest: matrix metalloproteinases in neuropsychiatric diseases, cardiovascular diseases, cancer, rheumatoid arthritis, wound healing, and periodontal diseases with their psychological implications, as reported below.

### 3.6. Matrix Metalloproteinases: Neuropsychiatric Diseases and Psychosocial Implications

In the field of neuropsychiatric diseases, four articles analyzed the molecular mechanisms, particularly those concerning the presence of MMPs, in schizophrenia [76], depression [77], autism spectrum disorders [78], and the auditory system in patients affected by Fragile X Syndrome [79], with particular attention to psychosocial implications.

In their study, Rahimi et al. [76] discovered that schizophrenic patients exhibited a significantly higher expression ratio of MMP-9/TIMP-1 (metallo-proteinase-1) compared to individuals without schizophrenia (SCZ). This finding underscores the importance of MMPs in contributing to the negative and positive symptoms commonly observed in schizophrenic patients, which can significantly affect their social functioning and daily life. MMP-9 has negative effects on depression, and a high expression of MMP-9 influences perineuronal net remodeling [77]. Perineuronal nets (PNNs) are a specialized extracellular matrix structure in the cortex, which increases the excitability of PV cells (parvalbumin-expressing interneurons) and increases inhibitory signaling pathways; PNNs can be degraded by the activity of MMP-9, causing abnormal auditory system responses in Fragile X Syndrome (FXS) [79]. Neuronal development and neuroplasticity are significantly influenced by MMPs, which can induce hyperplasticity, determining autism spectrum disorder (ASD), as shown by Abdallah et al. [78]. Most of the research in the field of autism analyzed the association of MMP levels with the risk of Fragile-X Syndrome (FXS), a disorder that affects 30% of patients with ASD [79]. Maintaining the blood–brain barrier (BBB) plays an important role in neuropsychiatric disorders. Inflammation is the key factor that initiates a cascade of events leading to cognitive decline and altered social interactions [76,77,78,79]. Within the context of autism spectrum disorder (ASD), MMPs have been identified as contributing factors to neuroinflammation and the pathological effects of infections. MMPs activate cytokines such as tumor necrosis factor α (TNF-α), interleukin 1β (IL-1β), and chemokines [78].

The regulation of neuronal cell differentiation in the central nervous system and monitoring of long-term potentiation (LTP) synaptic plasticity is highly dependent on maintaining a balanced MMP-9/TIP-1 ratio [76]. During depressive states, the conversion of brain-derived neurotrophic factor (BDNF) into its active form plays a crucial role in the late phase of LTP. This conversion is facilitated by extracellular proteases, such as MMP-9, which are responsible for remodeling synaptic connections [77]. In subjects with Fragile X Syndrome (FXS), MMP-9 is also involved in gamma asynchrony and cortical hyperexcitability of the auditory system [79]. The absence of FMRP (Fragile X Mental Retardation protein), an mRNA-binding protein that participates in key synaptic pathways, leads to an elevated level of MMP-9. Consequently, this results in reduced perineuronal nets (PNNs) surrounding parvalbumin (PV) cells and a subsequent decrease in the inhibitory effect of PV cells on cortical networks [79]. The significance of MMP-9 in the process of myelinogenesis and subsequent demyelination observed in cases of depression cannot be overstated. The activation of the sympathetic–adrenal medullary and hypothalamic–pituitary–adrenal axes is directly related to the association between the polymorphism of the MMP-9 gene and the state of depression in people experiencing chronic stress [77]. Chronic stress determines impaired social behavior and cognitive dysfunction, which are hallmarks of neuropsychiatric disorders [76,77]. In patients with schizophrenia, individual expression levels of MMP-9 were not statistically significant, except for the MMP-9/TIP-1 ratio [76], while high levels of MMP-9 are crucial for the pathogenesis of depression, ASD, and FXS [77,78,79]. From a therapeutic perspective, some MMP-9 inhibitors have been proposed for their pharmacological treatments [77,78,79]. Considering the substantial amount of evidence that indicates a direct link between the severity of depression and elevated MMP-9 levels, it is plausible that MMP-9 could be a unique biomarker for depression. However, it is important to note that blood levels of MMP-9 do not necessarily reflect MMP-9 levels in the brain [77].

### 3.7. Matrix Metalloproteinases: Cardiovascular Diseases and Psychosocial Implications

In the field of cardiovascular diseases (CVD), four articles studied the impact of psychosocial factors on acute coronary syndrome [80], on the risk of coronary artery disease [81], on myocardial infarction [82], and on cardiovascular health in general [83].

Machulsky et al. [80] found that a major cause of acute coronary syndrome (ACS) is atherosclerotic plaque rupture characterized by several processes, such as matrix degradation; therefore, MMPs determine plaque instability.

Research by Jonsson et al. [82] reveals that elevated levels of MMP-9 can be identified not only in atherosclerotic lesions but also in peripheral blood. Additionally, the study by Garvin et al. [81] demonstrates a correlation between psychosocial factors and circulating levels of MMP-9, regardless of comorbidities, traditional cardiovascular risk factors for CAD, and ongoing drug therapy. This association was observed in a group of middle-aged individuals with no abnormal health conditions.

The analysis conducted by Shafi et al. [83] explores the relationship between socioeconomic status (SEP) and cardiovascular health disparities via the examination of proteomic elements, identifies 184 biomarkers predictive of cardiovascular disease (CVD), and clarifies their implications for a better understanding of the etiology and evolution of CVD.

In individuals with ACS and limited social support, there is a positive correlation between hostility and MMP-2 activity, regardless of traditional risk factors. On the contrary, in individuals with high levels of hostility, IL-1β is associated with increased levels of MMP-9, indicating a connection between psychological factors and the inflammatory response [80]. The relationship between MMP-9 and the psychosocial context remains significant even after accounting for variables such as smoking, excessive alcohol consumption, poor diet, and physical inactivity. Furthermore, depression and cynicism independently contribute to elevated levels of MMP-9, as demonstrated by Garvin et al. [81]. While Jonsson et al. found a dysregulation of MMP-9 and TIMP in blood mononuclear cells from subjects who have experienced a myocardial infarction (MI), these factors may be unrelated phenomena [82]. Referring to other MMPs, such as MMP-12, Shafi et al. [83] showed that it is correlated with education by a strong negative association; in terms of lower values of MMP-12 and higher educational levels, MMP-12 and family income are also significantly correlated; smoking and MMP-12 are positively associated in terms of high MMP-12 values and high levels of smoking.

The findings indicate a significant role for MMP-9 in plaque degradation and the occurrence of acute cardiovascular events. However, no associations were observed between cortisol levels, hostility, social support, and perceived stress [80,81]. High levels of MMP-9 and depressive state are predictive but independent factors with a potentially strong impact on the cardiovascular prognosis after MI [82]. Socioeconomic position can be incorporated into the CVD risk prediction model by associating biomarkers from inflammatory pathways (such as MMP and IL-6), cell adhesion, platelet activation, blood pressure, and MAPK cascade with social phenomena [83].

### 3.8. Matrix Metalloproteinases: Cancer and Psychosocial Implications

In the context of biological pathways that have correlated MMPs and social aspects in cancer, our methodological approach allowed us to analyze four articles that focus on ovarian cancer in women [84,85,86,87], one article on lung adenocarcinoma [88], and one article on breast cancer [89]. The study by Lutgendorf et al. [84] focuses on the linking between behavioral distress and higher levels of angiogenic cytokines (i.e., interleukin-6) and MMPs produced by the tumor microenvironment of stromal cells of ovarian cancer. Thaker et al. [85] described neuroendocrine effects on cancer, how ovarian cancer progression is caused by stress responses, and β-adrenergic receptors that promote angiogenesis and tumor growth in ovarian cancer cells. A review by Sobhani et al. [86] described the link between stress and immune responses, with a special focus on cancer growth mechanisms, such as neovascularization, MMP activity, CREB activation and hypoxia-induced genes, DNA repair, DNA degradation, apoptosis, and inflammation. The influence of stress on tumor progression is discussed in the review by Eckerling et al. [87], while Gonzalez-Avila [88] analyzed MMP levels and stress hormones in patients with lung adenocarcinoma. In the study by Shi et al. [89], the authors explored the connections between symptoms, hematopoietic stem cells (HSCs), and cytokines in patients with breast cancer. This research reveals that fatigue and insomnia are related to elevated levels of IP-10 and TNF-RII in the blood, while mental fatigue is associated with elevated levels of MMP-2. These findings provide valuable information for future investigations into the underlying mechanisms and potential interventions for psychological symptoms of breast cancer.

Depressed patients and those with higher levels of chronic stress, current stress, and negative effects had increased expression of MMP-9 in tumor-associated macrophages (TAM). On the contrary, individuals with greater social support exhibited reduced levels of vascular endothelial growth factor (VEGF) and MMP-9 in tumor cells. In particular, MMP-2 expression in TAM did not show a significant correlation with the biobehavioral factors mentioned [84]. In their review, Thaker and Sobhani highlighted the role of MMPs in driving ovarian tumor progression via the promotion of angiogenesis. This is achieved by breaking down the extracellular matrix and facilitating the migration of endothelial cells, ultimately leading to angiogenesis [85,86]. MMP levels during angiogenesis are directly correlated with the abundance of signals and molecules that dictate tumor metastasis. In addition, extracellular matrix-derived angiogenic factors contributed further to this process [86]. Norepinephrine and adrenaline determine MMP production; stress is responsible for MMP secretion by stromal and tumor cells [87].

The articles [84,85,86,87] highlight the possibility that stress hormones may directly contribute to tumor cell progression via the modulation of MMPs. When isolated human macrophages were stimulated with norepinephrine and hydrocortisone in an in vitro setting, MMP-9 production increased. This suggests that in the context of behavioral disorders or low social support, elevated levels of stress hormones can lead to upregulation of MMP-9, MMP-2, and VEGF expression in ovarian tumor cells via β-adrenergic receptor signaling [84].

According to research conducted by Gonzalez-Avila [88], increased levels of MMPs and stress hormones were found in the blood. However, weak correlations were found between stress hormones and MMPs and TIMPs. The authors suggested that MMPs could potentially serve as indicators of lung cancer progression, but further research is needed to confirm this hypothesis.

Fatigue and insomnia are a common occurrence among breast cancer patients undergoing adjuvant radiation therapy (RT) and were associated with changes in interferon γ-induced protein 10 (IP-10) and tumor necrosis factor receptor II (TNF-RII), while mental fatigue was associated with increased levels of matrix metalloproteinases-2 (MMP-2) [89].

### 3.9. Matrix Metalloproteinases: Rheumatoid Arthritis and Psychosocial Implications

In the case of inflammatory diseases, four articles have studied the pathological mechanisms involved in MMP in rheumatoid arthritis (RA) associated with the psychosocial sphere [90,91].

Cohen et al. [90] investigate the factors that can predict the quality of life in individuals with rheumatoid arthritis (RA) over a period of 5 years. They accomplish this using Arthritis Impact Measurement Scales 2 (AIMS2) as a questionnaire designed to measure specific outcomes related to RA.

Vendrusculo et al. [91] conducted an observational cross-sectional study to provide a comprehensive description of chronic pain in Brazilian women with rheumatoid arthritis (RA) compared to women without RA or chronic pain, who will serve as the control group.

In the study by Durez et al. [92], the authors compared the short-term clinical and biological effects of intravenous pulse methylprednisolone (MP) and the tumor necrosis factor (TNF) antagonist infliximab (IFX) in patients with severe RA treated with methotrexate (MTX).

Murakami et al. [93] described the role of cytokines and MMPs in the progression of rheumatoid arthritis.

The AIMS2 proposed by Cohen consists of five domains, of which the social component was the only domain for which the Health Assessment Questionnaire (HAQ) score was not an independent predictive factor, but antiperinuclear antibodies, MMP-3 levels, joint space narrowing, and tender joint scores were predictive of social life [90].

According to Vendrusculo et al., collagenases MMP-2 and -9, which degrade collagens and cleave ECM proteins, play a crucial role in joint destruction and the development of pain during RA progression, and therefore their inhibitors may represent a new therapeutic treatment of pain in patients with rheumatoid arthritis [91].

Based on the analysis of the findings, it was determined that the level of MMP-3 can independently predict the quality of life of RA patients over a period of 5 years [90]. On the other hand, MMP-2 and MMP-9 are associated with pro-inflammatory cytokines, including IL-6, TNF-α, IL1-β, and cortisol [91]. Research papers [90,91] have demonstrated that blood biomarkers, such as MMPs, in conjunction with emotional support, play an important role in the clinical and therapeutic management of RA. These MMPs can accurately predict pain levels [91], improve the biopsychological well-being of patients, and ultimately contribute to improved quality of life [90]. Behavioral and psychosocial analyses have shown that women with RA and chronic pain have a worse quality of life (QoL) and willingness to live with pain, suggesting the need to monitor these women throughout their daily lives [91].

In the paper by Durez et al., the quality of life of patients was measured and serum levels of MMP-3 and IL-6 as inflammatory indices were assayed. MMP-3 levels decreased 6 weeks after IFX treatment, while no change was observed in the group of patients treated with MP [92]. Murakami et al. established that the destruction of joints in rheumatoid arthritis (RA) is attributed to the dysregulation of cytokines that determine the activation and differentiation of osteoclasts. Furthermore, TNF and IL-6 are involved in the production of MMPs that break down collagen and proteoglycan in cartilage and bone [93]. Oral glucocorticoid therapy, like MP, increases MMP-3 levels, and this also leads to the destruction of cartilage and the extracellular matrix of bone via the activation of proMMP-1 and proMMP-9. No scales that measure quality of life improved with MP treatment, while some improved in the case of subjects treated with IFX [92]. From Murakami’s article, it emerged that targeting cytokines with biological agents has shown positive outcomes, including clinical remission, protection against joint destruction, and improved quality of life (QOL) for patients with RA [93].

### 3.10. Matrix Metalloproteinases: Wound Healing, Periodontal Disease, and Psychosocial Implications

In the context of wounds, Vedhara et al. conducted a prospective observational study in which they evaluated salivary cortisol, MMP-2, and nine levels from a sample of 93 patients with diabetic foot ulcers, analyzing MMPs in the biological healing process, including confrontation, coping, and depression [94].

In the study by Kellis et al. [95], it was discussed that when the normal progression of wound healing is interrupted, a chronic wound emerges, creating an unfavorable environment characterized by increased levels of pro-inflammatory cytokines, increased matrix metalloproteinases (MMPs), the destruction of components of the extracellular matrix (ECM), and reduced activity of growth factors and other soluble mediators. In the article by Lobmann R. et al. [96], impaired wound healing is a significant issue in individuals with diabetes, often leading to non-healing ulcers. The presence of abundance levels of matrix metalloproteases (MMP) determines the chronicity of these wounds. To address this, the application of protease inhibitors topically may have a positive impact on a chronic wound, facilitating passage to an acute wound.

The findings indicate that people suffering from depression and lacking effective coping mechanisms experience a slower reduction in the ulcer area during the healing process. Furthermore, healed ulcers at 24 weeks are associated with lower levels of evening cortisol, elevated levels of MMP-2 precursor, and increased cortisol awakening [94]. Collagen, a vital component of the skin, plays a crucial role in adjunctive wound therapy by stimulating the recruitment of immune cells and fibroblasts. It sacrificially undergoes degradation by MMP, thus preserving the integrity of the native ECM and facilitating healing [95]. The study by Lobmann et al. [96] analyzed the implications of a protease inhibitor, applied topically, on the wound healing process and found that it had a beneficial effect. Interestingly, MMP mRNA levels remained unchanged during treatment with protease inhibitors, both at the wound fluid and at the cellular level. In patients with diabetic foot ulcers, MMP-2/-9 are linked to the healing process of dermal ulcers, and increased expression of these MMPs is correlated with ulcer chronicity; particularly, MMP-2 is considered the biomarker of connective tissue remodeling, and MMP-9 is the biomarker of inflammatory cell infiltration [94]. In the paper by Lobmann et al., a decrease in the MMP-9/TIMP-2 ratio emerged, which is a relevant indicator of wound healing. Additionally, despite the reduction in wound size, the authors observed no compensatory increase in MMP-RNA expression [96].

Schmidt et al. [97] conducted a study that examined the correlations among stress hormones, specifically cortisol and dehydroepiandrosterone-sulfate (DHEA-S), an inflammatory marker (IL-6), and an increased depth of periodontal examination (PD) in young people who presented initial indications of periodontal disease. In the study by Jung et al. [98], the presence of periodontal disease is observed in individuals diagnosed with systemic sclerosis (SSc). This study aimed to compare the surface area of the periodontal ligament (PDL) in patients with systemic sclerosis with that of a control group. The objective of the research conducted by Noack et al. [99] was to examine the correlation between the presence of active circulating MMP-8 (aMMP-8) and the status of periodontal disease, as well as the presence of aMMP-8 in oral fluid of individuals who are otherwise in good health. In children with periodontal disease, active MMP-8 is a potential tool for diagnosing periodontal inflammation, so in addition to measuring PD, a matrix metalloproteinase-8 test was performed from the saliva of the participants in the scientific work by Schmidt [97]. In the study by Jung et al. [98], an evaluation of quality of life and oral health was performed by measuring multiple biomarkers (IL-6, MMP-9, and CXCL-4) in gingival crevicular fluid (GCF) were conducted in the study by Jung et al. [98]. The GCF of patients with SSc exhibited significantly higher levels of IL-6, MMP-9, and CXCL-4 compared to the control group. Specifically, MMP-9 levels were found to be elevated only in the initial stage, indicating its potential as an early prognostic indicator for SSc [98]. Patients with periodontitis exhibited significantly higher levels of oral aMMP-8 compared to those who were periodontally healthy or had gingivitis. Furthermore, the highest levels of aMMP-8 in the bloodstream were observed in the group with periodontitis in the study conducted by Noack et al. [99].

The experimental study by Schmidt et al. [97] revealed that there was no correlation between stress-related hormones DHEA-S, cortisol, and maximum PD. However, it was suggested that a positive MMP-8 test could potentially be linked to higher levels of DHEA-S and BMI, indicating a possible relationship between psychological stress, patient diet, and the inflammatory process commonly observed in this disease. In the research by Noack et al. [99], aMMP-8 levels in the bloodstream showed a significant correlation with both oral aMMP-8 levels and clinical indicators, following a pattern that depended on the dose. After taking into account potential confounders, the concentrations of aMMP-8 in saliva, as well as the severity of periodontitis, were identified as significant predictors of aMMP-8 in the bloodstream.

Salivary cortisol is relevant because it measures the functionality of the hypothalamic–pituitary–adrenal axis (HPA), a system that primarily interconnects psychological distress and immunity [94]. Stress is also an etiological factor in periodontal disease, causing hyperactivity of the HPA axis, leading to the secretion of corticotrophin-releasing hormone (CRH) [97].

### 3.11. How MMPs Relate to Social Issues

In the context of neuropsychiatric disease, social aspects can be found when considering the manifestations of typical symptoms of these disorders [76,77,78,79]. In fact, in Rahimi’s study [76], MMP-9 and TIMP-1 are involved in neurotransmission processes and are strong candidates for the appearance of positive symptoms and negative symptoms, as well as cognitive dysfunction typical of schizophrenia. The study by Li Hogmin [77] also shows that MMP-9 is involved in the altered synaptic transmission mechanisms typical of depression symptoms that create psychosocial distress in affected individuals, making this disease very difficult to manage. In the reviews by Abdallah and Razak [78,79], MMPs play an important role in neuronal development; in particular, their influence is evident in abnormal connections between neurons and disrupted synaptic mechanisms [78]. These processes underlie the cognitive decline and social implications typical of ASD and FXS, such as social anxiety, hyperactivity, and hypersensitivity (referred to as abnormal sensory sensitivity typical of FXS) [79].

The “trait d’union” between MMP and psychosocial effects is a risk factor for cardiovascular disease [80,81,82,83]. Extensive research has been conducted on well-known risk factors for cardiovascular disease (CVD), including smoking, hypertension, diabetes, and dyslipidemia. However, it is important to note that these factors do not predict all clinical cases of CVD, as new risk factors also play a role in the development of the disease [80]. In the analyzed studies, it is very evident how hostility, depression, and work stress can influence the biological activity of MMP, contributing to CVD [80,81,82,83]. When evaluating cardiovascular risk, physicians typically focus on traditional risk factors, but these factors only provide a partial understanding of the challenges faced by people affected by cardiovascular disease. Other important aspects, such as socioeconomic status and its impact on mental health, are often overlooked [83].

In the field of rheumatoid arthritis (RA), MMPs are correlated with psychosocial effects in terms of predictive factors for the disease [90,91]. The progression of the disease consists of the following steps: pain, joint destruction, severe functional disability, deterioration of quality of life, and even death [90,92,93]. Predicting the outcome of RA is important because it allows us to identify pharmacological treatments that reduce disease severity and poor prognosis [91,92,93]. The reviewed articles identified possible biological prognostic factors for rheumatoid arthritis and considered quality of life, perception of pain, and acceptance of disability as the main objectives of the therapeutic strategy [90,91,92,93]. Psychological factors related to pain and anxiety experience are the basis for the progression of RA to the most severe form [91].

Chronic stress can induce tumorigenesis and promote the development of cancer and metastases; therefore, the management of stress and depression is essential for cancer patients [84,85,86,87,88,89]. When individuals experience behavioral distress or lack social support, there is an increase in the production of stress hormones such as catecholamines and glucocorticoids. This elevation in hormone levels can stimulate the production of MMP-9 by TAMs, creating a favorable environment for angiogenesis, invasion, and the production of VEGF, IL-6, and MMP-9 in patients with ovarian cancer and lung adenocarcinoma [84,88]. In the field of breast cancer, it has been observed that mental exhaustion, linked to elevated levels of MMP-2, plays a crucial role in determining the overall quality of life for people affected by this disease [89].

Stress is also responsible for the relationship between MMP and psychosocial implications in chronic wound healing and periodontal disease [94,95,96,97,98,99]. Vedhara et al. [94] examined the correlation between cortisol levels and MMPs, which play a crucial role in the development of chronic dermal ulcers, was examined by Vedhara et al. [94] via the analysis of saliva samples from patients with diabetic foot ulcers. Kallis et al. [95] analyze wound management as a social problem due to high costs and low quality of life for people with pressure ulcers, diabetic foot ulcers, and venous leg ulcers. Also, Lobmann et al. [96] regard the non-healing of diabetic foot ulcers as a complication with a considerable burden on the social life of individuals.

Stress is thought to be one of the triggers for the inflammatory processes typical of periodontal disease, so Schmidt et al. [97] showed the relationships between stress hormones (cortisol and DHEA-S), inflammatory processes (using the salivary MMP-8 test), and the health of young patients (oral health condition, eating habits, and socioeconomic status). The oral health and quality of life of patients with periodontitis emerged in the paper by Jung et al., and Noack et al. [98,99].

## 4. Discussion

The main findings of the present research revolve around the involvement of MMPs in the inflammatory processes underlying various human diseases and the “complex” dimension of MMPs, which, in addition to their biological significance, includes their psychosocial implications according to Morin’s “paradigm of complexity” [66]. In order to simplify the arguments presented, the discussion is divided into the following subsections.

### 4.1. The Inflammatory Function of Matrix Metalloproteinases in Human Diseases

As can be seen from the studies analyzed, the role that MMPs play in various pathological mechanisms has a negative impact on disease. The trigger for neuropsychiatric, cardiovascular, rheumatoid arthritis, wound healing, and periodontal diseases is the onset of an inflammatory process involving MMP [76,80,85,91,97].

In the field of neuropsychiatric disorders, the extracellular matrix in the CNS consists of the basement membrane, perineuronal nets, and the interstitial matrix between neural cells. MMPs can influence the integrity of the blood–brain barrier (BBB), and its breakdown depends on damaged endothelial cells that facilitate the permeability of inflammatory molecules [76]. The degradation of proteins found in the blood–brain barrier (BBB), specifically tight junction proteins and basal lamina proteins, is facilitated by MMPs. This degradation ultimately leads to leukocyte infiltration, cerebral edema, and hemorrhage [78]. The progression of neuropsychiatric diseases is primarily driven by inflammation and an exaggerated immune response, both of which are significantly influenced by the remodeling of the extracellular matrix via the actions of MMPs [100].

The secretion and maturation of pro-inflammatory cytokines, such as IL1b and TNFa, in schizophrenia (SCZ) may be facilitated by MMP-9 [101]. Additionally, impaired synaptic pruning, which is believed to contribute to SCZ development, may be caused by the phagocytic and proteolytic activity of MMP [102]. Furthermore, MMP-9 plays a role in the elimination of the receptor for advanced glycation end products (RAGE), a membrane glycoprotein that belongs to the immunoglobulin superfamily. The activation of nuclear factor kB (NF-kB) by RAGE leads to the expression of various inflammatory cytokines, which hinder the normal maturation of PV neurons in individuals with schizophrenia [103]. The involvement of MMP-mediated remodeling of the ECM has been suggested as a potential factor that contributes to the excitatory–inhibitory imbalance associated with depressive-like behavior in stress-based models of depression [104]. MMP-9 is more involved in the pathological mechanisms of depression; in fact, Domenici et al. [105] demonstrated a positive correlation between the severity of depression and high serum levels of MMP-9 in patients with major depressive disorder. In their study, Bobi’nska et al. [106] investigate the presence of MMPs (MMP-9, MMP-2, and MMP-7) and TIMP-2 in the blood of people experiencing depression. A pilot study conducted by Chandrasekaran et al. [107] demonstrated, as its main finding, elevated serum levels of MMP-9 in patients diagnosed with bipolar disorder and a significant duration of suicidal thoughts.

In recent decades, there has been a significant improvement in understanding the role of MMPs in the neurobiology of ASD, with a particular focus on Fragile X Syndrome, the most common complication in patients with ASD manifested by hyperactivity, impulsivity, anxiety, poor language development, and seizures [108]. The pro-inflammatory effects of MMP have been found to cause abnormalities in individuals with ASD, leading to disruptions in the blood–brain barrier (BBB) and exposing the central nervous system (CNS) to external factors and infiltrating cells [109]. MMP-9 plays a role in amplifying the inflammatory response by activating tumor necrosis factor-alpha (TNF-α), which, in turn, triggers MMP-9 expression via the expression of MMP-9 through TNFR1/TRAF2/PKC-α-dependent pathways, creating a positive feedback loop [110]. Furthermore, MMPs have been implicated in neuropsychiatric disorders, where they contribute to a phenomenon known as “toxic gain of function” or “loss of function/dysfunction”. Specifically, elevated levels of MMP lead to increased pro-inflammatory cytokine signaling, disruption of BBB homeostasis, and significant degradation of PNNs, making neurons more vulnerable to degenerative changes in the redox and ionic potential of the extracellular environment [100].

For CVD, MMP-9 can play a direct role as a biomarker of plaque vulnerability, can be elevated in patients diagnosed with angina or myocardial infarction, is associated with cardiovascular risk factors before the onset of the disease, and is upregulated by pro-inflammatory cytokines, suggesting its impact on the inflammatory process [81]. In the process of inflammation that triggers the formation of atherosclerotic plaque, paracrine substances released by smooth muscle cells and leukocytes contribute to the degradation of the extracellular matrix, which ultimately leads to plaque rupture [111]. This rupture is responsible for the appearance of adverse events such as stroke and myocardial infarction. High levels of MMP-2 and MMP-9 have been implicated in the pathological events mentioned above [103]. Specifically, MMP-9 directly degrades ECM proteins via the activation of cytokines and chemokines, which are involved in the regulation of tissue remodeling [112]. A study of 53 male patients with coronary artery stenosis and 133 subjects without cardiovascular disease examined the levels of MMPs in their plasma. The findings revealed that patients with CAD had markedly elevated levels of MMP-9, while levels of MMP-2 and MMP-3 were lower [113]. In another study, plasma levels of MMP-9 and TIMP-1 were compared between patients with left anterior descending artery lesions and healthy individuals. The results demonstrated an increase in plasma levels of MMP-9 and TIMP-1 during acute coronary syndromes [114].

In cancer, MMPs, along with cytokines, growth factors, chemokines, and enzymes, help stimulate inflammatory events that increase the risk of cancer [115]. Tumor metastasis depends on the degradation of ECM by MMPs, which also expose their binding sites to other receptors by releasing a bioactive molecule [116]. In scientific research, the biological role of MMPs in ovarian cancer has attracted a great deal of attention. Indeed, an interesting review article analyzes MMPs in different types of ovarian cancer and reflects on their importance in tumor progression. Tumor growth, invasion, angiogenesis, and, ultimately, metastasis are promoted by activating MMPs released from ovarian cancer cells and surrounding stromal cells [117,118]. In the case of lung cancer, several studies support the hypothesis that MMPs participate in the inflammatory processes that make lung tissue malignant [119]. In addition to their role as tissue biomarkers, MMPs, specifically MMP-2 and -9, have been extensively studied as potential serum biomarkers for the prognosis of breast cancer. These secreted MMPs have the ability to degrade the basement membrane, allowing cancer cells to invade the surrounding stroma and promoting cancer progression. Furthermore, MMPs can directly impact tumor cells by releasing factors that stimulate growth or inhibit apoptosis. In addition, MMPs have been found to induce phenotypic changes in association with the epithelial–mesenchymal transition, a developmental process that becomes activated during tumor progression [120]. Research conducted by Mittal et al. revealed that the complexity of the role of each MMP in the evolution of cancer is greater than expected. This suggests the need for additional research on this topic [121].

The pathophysiology of rheumatoid arthritis (RA) involves the contribution of cytokines, specifically TNF-α, IL1-β, IL-6, and IL-8, which play significant roles in the process of synovial inflammation and joint destruction by inducing MMP expression [91]. MMPs primarily regulate bone destruction via various mechanisms, including collagen degradation and cartilage damage by adhering to chondrocytes, as well as the regulation of inflammatory cytokines and chemokines, leading to the activation of inflammatory signaling pathways and facilitating cell migration and invasive angiogenesis [122]. TNF has been observed that TNFα triggers the expression of MMP-2, -3, -8, and -9 via a signaling cascade involving mitogen-activated protein kinase (MAPK) and protein kinase C (PKC), highlighting the association between cytokines and MMPs in the inflammatory process of RA [123]. The main functions of MMPs in the wound healing of chronic wounds, such as pressure ulcers, diabetic foot ulcers, and venous leg ulcers, are distinguished into several stages: the inflammatory stage characterized by the degradation of ECM, the proliferative stage that promotes angiogenesis and cell migration, and the remodeling stage in which tissue remodeling takes place [124,125]. The role of MMPs in wound healing is crucial, as they play a significant role in every stage of the process. However, in the case of diabetic foot ulcers (DFU), the excessive expression of MMP can result in excessive tissue degradation and unregulated activity of these proteases, ultimately leading to nonhealing of wounds [126]. An analysis of various studies reveals the contrasting characteristics of foot ulcers that have healed versus those that have not. Non-healed ulcers exhibit elevated levels of MMP-1, -2, -8, and -9, while showing lower levels of TIMP-1. This disparity in the ratio between MMPs and TIMP-1 contributes to the development of chronic ulcers, and a deficiency in the expression of MMPs can hinder the proper and timely healing of ulcers [127].

Changes in MMP levels contribute to the development of oral diseases and play a role in the regeneration of natural tissues and destructive processes associated with periodontal disease. Furthermore, MMPs can serve as a potential risk factor for periodontal disease and as biomarkers for the early detection of periodontitis, making them a valuable tool for monitoring the prognosis [128]. Collagenases such as MMP-8 and MMP-13, since type I collagen is involved in the composition of the periodontal extracellular matrix, and gelatinases such as MMP-2 and MMP-9 exert a determinant role. In particular, MMP-8 can be analyzed from saliva or in gingival crevicular fluid [129]. MMP-8 and MMP-9 are the most abundant in periodontal tissues, and the presence of polymorphonuclear leukocytes, macrophages, plasma, fibroblasts, endothelial cells, keratinocytes, and bone cells in oral fluids is responsible for the observed secretion [130].

Although there is evidence in the literature for the negative effects of MMPs in chronic and inflammatory human diseases, consistent with the results of our review, they should not be classified in a simplistic manner. In fact, its role in disease could also be positive, but further studies are needed to clarify this. In certain cases, research studies focused on schizophrenia have shown that there are no notable variations in the expression of MMP-9 in blood samples collected from individuals diagnosed with SCZ compared to those without the condition [76,131].

### 4.2. Matrix Metalloproteinases and Psychosocial Implications in Chronic and Complex Diseases

The results of the analysis of the article in this current study showed an important correlation between MMPs in human disease and psychosocial aspects, which represent a very heavy burden in the lives of affected people.

The state of mental health encompasses an individual’s ability to effectively manage and cope with the typical stressors that arise in life, promoting a sense of overall well-being; it determines how we think, feel, and act psychologically, emotionally, and socially. One’s biological structure (genes, neurotransmitters, etc.) can influence the state of mental well-being, and when individuals lose mental balance, mental disorders appear, and the relationship with others, perceptions, emotions, and thoughts are altered [132].

In schizophrenia, MMPs have been associated with positive and negative symptoms and cognitive dysfunction with a profound impact on patients [100]. In a study conducted by Gao et al., it was demonstrated that the level of DNA methylation at GpG sites within the MMP-9 gene in peripheral blood mononuclear cells exhibited an inverse correlation with negative symptoms of schizophrenia, particularly social motivation [133].

The hypothesis surrounding SCZ suggests that brain development can be impacted by various factors, including prenatal, genetic, and environmental influences. These factors can make the brain more susceptible to events such as stress, increasing the likelihood of experiencing symptoms in adulthood [134]. The study by Bitanihirwe emphasizes the significance of MMP-9 in relation to this hypothesis [102].

Abnormal synaptic connections disrupt neuronal pathways, and the insufficient pruning of the synapse determines the symptoms of ASD that cause social problems for the affected individual. MMPs exert an important function in neuroplasticity, synaptic changes, and neuronal growth [135]. A case–control study showed a positive association between the rs3918242 MMP-9 polymorphism (-1562C/T) and increased serum MMP-9 levels in patients with AD, and finally, the authors propose the TT genotype as a genetic risk factor [136]. Extensive research has been conducted on genetic modification associated with autism spectrum disorder (ASD), specifically focusing on FMR1, the gene responsible for producing fragile X mental retardation protein (FMRP) [79]. The absence of FMRP results in fragile X syndrome (FXS), a condition in which MMP-9, a protein, is found to be elevated in the blood of FXS patients. Interestingly, the administration of minocycline, a drug that decreases MMP-9 levels, resulted in noticeable improvements in the clinical condition of these patients [137].

Depression is a mental disorder that affects an increasing number of people and therefore represents a major social problem [138]. MMP-9 levels were found to be correlated with the severity of depression and quality of life in patients, as reported by Yoshida [139]. Furthermore, some studies have provided evidence of increased MMP activity leading to degenerative changes in response to stressful conditions [140]. Furthermore, Van der Kooji et al. discovered that increased MMP-9 activity in the CA1 hippocampus results in a reduction in the perisynaptic protein nectin-3. They also established that this effect of MMP-9 is dependent on excitotoxic stimuli from NMDA and is responsible for the behavioral consequences associated with chronic stress [141].

The primary cause of mortality in the world is cardiovascular disease (CVD), and various psychosocial factors, including stress, adversity, socioeconomic status, depression, and anxiety, have been linked to general health, specifically cardiovascular health. These psychological factors can impact the development of CVD via complex and diverse pathological mechanisms, in which MMPs play an important role [142]. In the study by Lundberg et al., it was shown that the presence of stress triggers the secretion of MMP-9, which in turn has an impact on the characteristics of individuals suffering from coronary artery disease [143]. Similarly, the research conducted by Szymanowski highlights the importance of MMP-9 as a potential connection between stress and cardiovascular disease. A dysfunctional cortisol response was associated with a failure to regulate systemic MMP-9 levels in CAD patients compared to controls; patients showed a lower cortisol response to stress. MMP-9 levels were significantly reduced in controls but remained unchanged in patients after stress [144]. The connection between psychosocial factors and cardiovascular health is undeniable. Negative experiences and stress can lead to behaviors that increase the risk of cardiovascular disease, such as smoking, substance abuse, an unhealthy diet, and a sedentary lifestyle. Furthermore, people with poor psychosocial health can face barriers to accessing health care. It is crucial to recognize psychosocial factors as risk factors in order to prevent cardiovascular disease, provide appropriate treatment for those already affected, and improve both psychosocial well-being and cardiovascular health [142].

The prevalence of anxiety and depression is high among individuals with RA. A study was conducted to explore the correlation between psychological factors and disease severity indicators in RA patients. The findings revealed that anxiety and depression were associated with increased levels of pain, fatigue, physical disability, and medical expenses. In addition, they were associated with a decrease in overall quality of life related to RA [145]. The intricate relationship between MMPs and biological and social aspects is of significant importance in RA. A narrative review examined the impact of mental health on the progression of RA, particularly in terms of patient-reported outcomes (PROs). Chronic inflammation, where MMPs serve as predictive factors, influences stress responses and effective coping mechanisms, leading to depression and poorer long-term outcomes in RA [146].

Long-term chronic stress accelerates tumorigenesis and tumor progression and leads to worse clinical outcomes in cancer patients, particularly those with ovarian cancer, lung adenocarcinoma, and breast cancer, as shown in the results of this review. It is clear that the best-known mechanisms of stress-related tumorigenesis are hypothalamic–pituitary–adrenal (HPA) axis dysfunction and involvement of the sympathetic nervous system (SNS) [147]. The neuroendocrine system is abnormally activated, and stress-related hormones contribute to increased expression of oncogenes, exacerbating chronic inflammation and immune function and involving MMPs [147,148]. These findings suggest complex interconnections between MMPs and stress in the pathogenesis of cancer [147,149]. The psychological impact of stress on patients is the greatest in the case of cancer, whatever its type [65]. Patients are stressed by the seriousness and uncertainty of the disease, physical difficulties, drug treatment, and the family environment. Stress contributes to worsening anxiety and depression, which reduces the quality of life of the cancer patient. Finally, holistic treatment, both medical and psychological, is proposed as the only ameliorative approach [150]. Feelings of guilt and shame can arise from the societal stigma associated with mental illness and specific forms of cancer, such as lung cancer. These negative emotions have the potential to trigger depression. An example where this is evident is the connection between smoking and lung cancer, which can cause patients to blame themselves for their condition and face social stigma if they have a history of smoking [151]. It is crucial to prioritize research on the potential long-term consequences of cancer treatments on mental well-being and prevention strategies. Various factors, including tumor-related attributes, treatment methods, individual psychological factors, social influences, and contextual elements, may all play a role in the emergence of depression and anxiety in individuals undergoing cancer treatment [152].

The coexistence of diabetes and impaired wound healing, along with other vascular complications, poses a significant public health challenge. These complications have the potential to lead to the development of chronic foot ulcers and the need for amputation. An imbalance in MMPs can contribute to the prolonged healing process often observed in people with diabetes [153]. More generally, more evidence suggests that the formation of a chronic wound places a significant psychological burden on patients, leading to a decline in their quality of life and elevated levels of stress [154]. The production of glucocorticoids, which is known to increase during times of increased stress, has been found to have a negative impact on the production of healing cytokines such as IL1α, IL1β, and TNFα. Additionally, in acute wounds, there is an overregulation of MMPs during periods of increased stress. Stress is associated with unhealthy behaviors that are not directly related to the wound healing process but may still contribute to the effects of stress on wound healing [155].

The presence of salivary films plays a crucial role in maintaining oral well-being and managing the balance of microorganisms in the mouth. Saliva contains both confirmed and potential biomarkers for various diseases derived from epithelial cells, neutrophils, the oral microbiome, gingival crevicular fluid, and serum. One notable example is the use of salivary cortisol levels as an indicator of stress, while MMP-8 and -9 show promise as markers for caries and periodontal disease [156]. As the presence of MMP-8 in oral fluid and serum was detected in patients with periodontitis, MMP-8 has been proposed as a sensitive biomarker, health indicator, and biomarker for response to drug treatment, such as doxycycline as an MMP inhibitor [157]. Psychological stress influences behavioral changes such as smoking, poor oral hygiene, poor compliance (responsible for increased bacterial infections), overheating, and a high-fat diet (due to which cortisol levels increase); these factors together determine the onset of periodontal disease [158].

There are many studies in the literature that agree with our results, but there are also studies that show a weak and random correlation between illnesses and psychosocial factors, so more research is definitely needed to obtain definitive results [159].

### 4.3. Matrix Metalloproteinases, Psychosocial Implications, and the Paradigm of Complexity

The intricate interaction between MMPs and psychosocial factors in each of the diseases examined highlights the need for a comprehensive perspective on the disease. This perspective should encompass the biological, social, environmental, and psychological aspects, using an interdisciplinary and integrated approach that prioritizes the overall well-being of individuals, including both their physical and social health [65].

Examination of the length of the telomere in an African American population provides a compelling illustration of how sociology, molecular biology, and human disease intersect. This research reveals that African Americans generally possess longer telomeres, resulting in an increased susceptibility to cancer. Using telomeres as a practical resource, we have the potential to improve cancer diagnosis and prevention, thus addressing the pressing social concern of ethnic and racial health disparities [160].

The paradigm of complexity helps us to understand the world in which we live, which is made up of dynamic systems interconnected between them (in fact, a system is a set of elements in relation). By enabling us to explore the unpredictable, chaotic, nonlinear, and uncontrollable behaviors of living systems, it provides us with valuable insights [161]. However, there is little use of the complexity paradigm in health research [162]. The complexity paradigm is based on concepts of intradisciplinary and transdisciplinary; it represents another way of conceptualizing knowledge; it is still under construction; and it is not reductionist but provides innovative bases to know and study problems. It derives from systems theory, computer science, and cybernetics and overcomes traditional knowledge, positivism, Newtonian physics, and symmetric mathematics. It is based on the social determinants of health and organizational culture in which health systems are considered complex adaptive systems [161,163].

This concept of complexity as a new approach to the study of pathological mechanisms of diseases has very relevant theoretical implications for our study. Psychosocial conditions, such as socioeconomic status, social support and networks, work stress, unemployment and retirement, social cohesion, and social capital, as well as religious beliefs, have diverse impacts on both health and disease states [162]. These factors affect morbidity and mortality directly via physiological processes and indirectly via behavioral pathways. In the past, there was a distinction between psychological illness with little biological basis and purely physical disease; today, we realize that in almost all cases, this clear separation does not exist [164].

In addition to theoretical implications, some practical implications can be deduced: the importance of MMPs as biomarkers in cardiovascular disease and cancer, for example, as shown by several studies where MMPs may predict some complications in patients, leading to personalized treatment and follow-up [165,166]. In the study by Shin et al. [167] the authors used a machine learning approach involving both biomarkers and “sociomarkers” to identify asthmatic patients at risk of hospitalization after a first visit.

They then compared the traditional risk model based on biomarkers and the risk model based on “sociomarkers” using an integrated database. The findings indicated that the presence of “sociomarkers” contributes significantly to the prediction of health outcomes, specifically in pediatric asthma cases [167]. Therefore, it is desirable that, in the near future, it will be possible to use “sociomarkers”, measurable indicators of a patient’s social state, in clinical practice, alongside biomarkers, providing health professionals with a valuable tool to learn about the disease, its characteristics, its evolution, and its response to treatment. Furthermore, it is important to address these issues in research and then translate them into concrete clinical applications aimed at the overall well-being of patients, using the paradigm of complexity as a new methodological approach in health systems [65,162,167,168].

### 4.4. Strengths and Limitations of the Research

The first limitation of this paper is the exclusion of a non-English paper and papers in which the study was conducted on animals, thus losing information. One notable strength of the study was the use of a comprehensive and scientifically rigorous search strategy that involved the use of the three main databases. Furthermore, the researchers employed the PICOTS method to establish clear inclusion and exclusion criteria. As a result, the number of selected articles was relatively low, which can be considered a limitation, particularly in the field of oncological diseases. The methodological approach with inclusion and exclusion criteria imposed certain restrictions on the scope of the research, focusing primarily on ovarian, lung, and breast cancer. However, it is important to note that the objective of this review was to provide a comprehensive overview of scientific evidence on the role of MMPs in human diseases and their psychosocial impact on patients. Therefore, all relevant articles were included to ensure a complete understanding of the relationship between MMPs and human diseases, as well as their social implications. An important strength is that, to our knowledge, there are no Italian studies among the selected papers, so our work could be pioneering in this field. The included papers come from different countries of the world, mainly America, Germany, Sweden, and France (Europe). The field of social and behavioral sciences has played a crucial role in reducing the occurrence of risk factors among adults in the United States, a country that is particularly attentive to this matter [169]. An important strength of this review is the diversity of research fields, from psychology to neuroscience, oncology, endocrinology, cardiology, and dentistry, so that we were able to cover the topic from a neuropsychiatric, cardiovascular, tumor, inflammatory immune (rheumatoid arthritis), periodontal, and diabetic foot wound healing perspective. Finally, a limitation is that the articles reviewed cover a fairly long period (20 years of research), but there are few articles in this area up to the years 2021–2022 when there was a greater production of articles dealing with our research question. These results encourage future research on this topic.

## 5. Conclusions

This study provides an accurate overview of a very specific and original topic: the role of metalloproteinases and their links with the psychological and social dimension of people suffering from neuropsychiatric, cardiovascular, cancer, rheumatoid arthritis, periodontal, and chronic wound diseases. The biological functions of metalloproteinases and their negative connotations at the level of molecular mechanisms that determine the onset and progression of disease were discussed. In almost all cases analyzed, increased expression of MP leads to destruction of the ECM and alteration of the cellular balance by inflammatory processes [76,80,85,88,90]. The relationship between MPs and the psychosocial aspects of different diseases has been discussed. Psychosocial aspects can be found in the manifestation of symptoms of neuropsychiatric diseases [76,77,78,79], in risk factors for cardiovascular disease [80,81,82,83], e.g., depression and the socioeconomic situation of patients, in predictive factors for complications of rheumatoid arthritis [87,88], in stress, as a trigger for pathological processes underlying cancer [84,85,86], periodontal diseases [90] and the healing process of diabetic foot ulcers [89]. Given the complexity of the issue, a multidisciplinary and complex approach is required, based on the model proposed by Edgar Morin [66,67,68], which can explain the importance of a global view of human health, both physical and psychosocial, combining medical science with human sciences. There is no health or disease that is not influenced by the psychosocial context of the individual [65,147]. More than 60 years of research on MMPs has led to their application in the clinical setting as a tool to improve disease risk management and to make therapeutic choices more selective [150]. In addition to MMPs as biomarkers, it is hoped that in the near future, there will be social markers in mathematical predictive models to provide complete knowledge of diseases, combining their physical and material aspects with the psychological and social status of patients [66,67,68,152]. In conclusion, this topic requires further research, in addition to our contribution, to better understand the type of relationships between MPs with psychosocial aspects as determinants of health via complexity science, which still needs more knowledge for future applications in the clinical field. This current review has an innovative and original character and paves the way for numerous future studies.

## Figures and Tables

**Table 1:** Scientific databases, keyword search strategy, and number of articles extracted.

Keywords	Scopus	Web of Science	PubMed
Metalloproteinase * and social *	324	875	319
Metalloproteinase * and social aspect *	40	15	43
Metalloproteinase * and social determinant * of health	56	209	8450
Metalloproteinase * and psychosocial factor *	38	26	909

* represents any number of characters, even zero.

**Table 2:** Criteria for the inclusion/exclusion of articles according to a modified PICOTS.

Parameter	Inclusion Criteria	Exclusion Criteria
Patient	Human patients	Non-human patients
Intervention	To analyze the relationship between MMPs, disease, and psychosocial aspects	To analyze only aspects of MMPs (biological, medical, etc.) without the relationship with disease and psychosocial impacts
Comparator	None	None
Outcomes	To underline the interconnection between MMPs and the psychosocial sphere with certain results.	Models, articles, or tools not focused on the complex relationship between MMPs, diseases, and psychosocial aspects
Timeframe	Unrestricted (Final extraction: October 2023)	Unrestricted (Final extraction: October 2023)
Study Type	Research articles and review articles published in academic journals	Book chapters, book reviews, vignette studies, supplements, study protocols, commentaries, guidelines, editorials, book, meeting abstract, letter to editors
Language	English	Non-English

The parameter setting was adapted “to “Study type” and included “Language”.

**Table 3:** General characteristics of the included studies.

Authors	Title	Year	Country	Methods/Study Design	Population	Study Content
Rahimi S. et al. [76]	Blood evaluation of the expression levels of matrix metalloproteinase 9 (MMP9) and its natural inhibitor, TIMP1 genes in Iranian schizophrenic patients	2017	Iran	Experimental study	50 patients with schizophrenia and 50 healthy controls	The MMP9/TIMP1 expression ratio of MMP9/TIP1 is significantly higher in schizophrenic patients compared with healthy subjects
Li H. et al. [77]	Matrix metalloprotein ase-9 as an important contributor to the pathophysiology of depression	2022	China	Systematic review	None	To review the literature characterizing the negative effect of MMP-9 on depression
Abdallah M.W. et al. [78]	Matrix metalloproteinases in autism spectrum disorders	2013	Germany	Systematic review	None	To focus on the mechanisms through which MMPs can contribute to autism spectrum disorder
Razak K.A. et al. [79]	Neural Correlates of Auditory Hypersensitivity in Fragile X Syndrome	2021	USA	Systematic review	None	To examine the etiopathological mechanisms of the auditory system in Fragile X Syndrome, a genetic factor linked to behavior associated with autism spectrum disorders
Fernandez Machulsky N. et al. [80]	Matrix metalloproteinases and psychosocial factors in patients with acute coronary syndrome patients	2016	Argentina	Prospective study	76 patients after angioplasty	To examine the relationships between hostility, perceived stress, and social support and their impact on MMP-2 and MMP-9 activity, pro-inflammatory cytokines, and hemodynamic factors in patients diagnosed with acute coronary syndrome
Garvin P. et al. [81]	Plasma levels of matrix metalloproteinase-9 are independently associated with psychosocial factors in a middle-aged normal population	2009	Sweden	Prospective study	402 participants from a normal population	To examine the potential correlation between psychosocial factors and circulating levels of MMP-9 while considering comorbidities and traditional risk factors for cardiovascular disease
Jonsson S. et al. [82]	Overexpression of MMP-9 and its inhibitors in blood mononuclear cells after myocardial infarction—Is It Associated with Depressive Symptomatology?	2014	Sweden	Observational study	57 patients after myocardial infarction and 41 healthy controls	To examine the potential link between levels of MMP-9 and depressive symptoms in a middle-aged normal population from Sweden following a myocardial infarction
Shafi B.H. et al. [83]	Socioeconomic disparity in cardiovascular disease: Possible biological pathways based on a proteomic approach	2022	Denmark	Experimental study	1142 participants derived from the Copenhagen Heart Study	To analyze the social inequality in cardiovascular health by linking proteomic factors to socioeconomic position (SEP) in patients with cardiovascular disease
Lutgendorf S.K. et al. [84]	Biobehavioral Influences on Matrix Metalloproteinase Expression in Ovarian Carcinoma	2008	USA	Experimental study	56 ovarian cancer patients	To focus on the link between behavioral distress and higher levels of MMPs produced by ovarian cancer
Thaker P.H. et al. [85]	The neuroendocrine impact of chronic stress on cancer	2007	USA	Systematic review	None	Neuroendocrine influence on cancer, how ovarian cancer progression results from the stress response, and the mechanisms by which β-adrenergic receptors promote tumor growth on ovarian cancer cells
Sobhani M.E. et al. [86]	A review on biomolecular basis of the role of psychological stress in cancer development and progres-sion of cancer	2010	Bangladesh	Systematic review	None	Review on the role of psychological stress and immune response in the progression of cancer
Eckerling A. et al. [87]	Stress and cancer: mechanisms, significance and future directions	2021	Israel	Systematic review	None	To describe the mechanisms that determine the correlation between cancer and stress
Gonzalez-Avila et al. [88]	Matrix Metalloproteinases and Stress Hormones in Lung Cancer Progression	2022	Mexico	Observational study	104 patients with lung adenocarcinoma	To determine blood levels of MMPs and stress hormones in lung adenocarcinoma patients
Shi W. et al. [89]	Inflammatory Biomarkers, Hematopoietic Stem Cells, and symptoms in Breast Cancer Patients Undergoing Adjuvant Radiation Therapy	2020	USA	Longitudinal study	147 patients with a diagnosis of breast cancer	Evaluation of psychological symptoms, cytokines, and MMPs in patients with breast cancer
Cohen J.D. et al. [90]	Health assessment questionnaire score is the best predictor of 5-year quality of life in early rheumatoid arthritis	2006	France	Prospective study	191 patients followed over 5 years	The baseline health assessment questionnaire score proved to be the most accurate predictor of the 5-year quality of life in patients with early rheumatoid arthritis
Vendrusculo-Fangel L.M. et al. [91]	Structural equation modeling provides insights into understanding the construct of chronic pain in women with rheumatoid arthritis	2021	Brazil	Observational and cross-sectional study	42 women with rheumatoid arthritis (RA) and 42 women without RA	To describe the relationships between RA disease and biopsychosocial aspects: acceptance of pain and quality of life in women with RA
Durez P. et al. [92]	A randomised comparative study of the short-term clinical and biological effects of intravenous pulse methylprednisolone and infliximab in patients with active rheumatoid arthritis despite methotrexate treatment	2003	Belgium	Randomized comparative study	27 patients with severe RA	Comparison of treatment with methylprednisolone and infliximab for patients with rheumatoid arthritis to improve their quality of life
Murakami M. et al. [93]	Inflammatory cytokines in rheumatoid arthritis	2012	Japan	Review	None	Analysis of cytokines and MMPs involved in the progression of rheumatoid arthritis
Vedhara K. et al. [94]	Coping style and depression influence the healing of diabetic foot ulcers: observational and mechanistic evidence	2010	UK	Prospective and observational study	93 patients with diabetic foot ulcer	In order to comprehend the impact of psychological distress on the healing process of diabetic foot ulcers, it is essential to analyze the biological mechanisms that influence the healing process
Kellis P.J. et al. [95]	Collagen Powder in Wound Healing	2018	Washington	Review	None	To discuss the use of collagen in the healing process of chronic wounds, which represents a problem in their management due to high costs, low quality of life, and significant morbidity and mortality
Lobmann R. et al. [96]	Expression of matrix metalloproteinases and growth factors in diabetic foot wounds treated with a protease absorbent dressing	2006	Germany	Observational study	33 patients with chronic diabetic foot ulcer	To analyze the effects of a protease inhibitor in chronic diabetic foot ulcer
Schmidt J. et al. [97]	Stress-related hormones in association with periodontal disease in adolescents results of the epidemiologic LIFE Child study	2019	Germany	Observational study	498 adolescents with early signs of periodontal disease	To examine the correlation between stress-related hormone levels, inflammatory markers, and early indicators of periodontal disease in children and adolescents.
Jung S. [98]	Influence of systemic sclerosis on periodontal health: A case-control study	2023	France	case–control study	39 patients and 30 controls	Evaluation of oral health and quality of life in patients with systemic sclerosis
Noack B. et al. [99]	Association between serum and oral matrix metalloproteinase-8 levels and periodontal health status	2017	Germany	Cross-sectional study	59 subjects: 19 healthy controls, 20 with gingivitis, and 20 with periodontal disease	Correlation between serum aMMP-9, oral MMP-8, and periodontal health

**Table 4:** Journal distribution and research areas of the included studies.

Name of Journal	Number of Articles	Research Areas
*ATHEROSCLEROSIS*	1	Cardiovascular system and cardiology
*CELL CYCLE*	1	Cell biology
*CLINICAL CANCER RESEARCH*	1	Oncology
*CLINICAL ORAL INVESTIGATIONS*	1	Dentistry, oral surgery, and medicine
*DIABETOLOGIA*	1	Endocrinology and metabolism
*FRONTIERS IN NEUROLOGY*	1	Neuroscience and neurology
*FRONTIERS IN PSYCHIATRY*	1	Psychiatry
*JOURNAL OF MOLECULAR PSYCHIATRY*	1	Psychiatry and neuropsychiatry
*MAGAZINE OF EUROPEAN MEDICAL ONCOLOGY*	1	Oncology
*METABOLIC BRAIN DISEASE*	1	Endocrinology and metabolism; neuroscience and neurology
*MODERN RHEUMATOLOGY*	1	Rheumatology
*PLOS ONE*	1	Cardiology
*PSYCHONEUROENDOCRINOLOGY*	1	Endocrinology and metabolism; neuroscience & neurology
*PSYCHOSOMATIC MEDICINE*	1	Psychiatry and psychology
*THE JOURNAL OF RHEUMATOLOGY*	1	Rheumatology
*NATURE REVIEW CANCER*	1	Oncology
*JOURNAL OF ONCOLOGY*	1	Oncology
*JNCI CANCER SPECTRUM*	1	Oncology and cancer
*ANNALS OF RHEUMATIC DISEASE*	1	Rheumatology
*CLINICAL CALCIUM*	1	Rheumatology and osteoporosis
*JOURNAL OF DRUGS IN DERMATOLOGY*	1	Dermatology and wound healing
*JOURNAL OF DIABETES AND ITS COMPLICATIONS*	1	Diabetes, wound healing
*JOURNAL OF CLINICAL PERIODONTOLOGY*	1	Periodontal disease
*JOURNAL OF PERIODONTAL DISEASE*	1	Periodontal disease
